# Nutrient Metabolism Pathways Analysis and Key Candidate Genes Identification Corresponding to Cadmium Stress in Buckwheat through Multiomics Analysis

**DOI:** 10.3390/genes14071462

**Published:** 2023-07-18

**Authors:** Dengxiang Du, Hanxian Xiong, Congping Xu, Wanyong Zeng, Jinhua Li, Guoqing Dong

**Affiliations:** School of Life Science and Technology, Wuhan Polytechnic University, Wuhan 430023, China; ddx@whpu.edu.cn (D.D.); xionghx@163.com (H.X.); foreverxcp@126.com (C.X.); vyzeng@163.com (W.Z.); ljh4431_cn@sina.com (J.L.)

**Keywords:** buckwheat, cadmium, transcriptome, metabolomics

## Abstract

*Fagopylum tatarium* (L.) Gaertn (buckwheat) can be used both as medicine and food and is also an important food crop in barren areas and has great economic value. Exploring the molecular mechanisms of the response to cadmium (Cd) stress can provide the theoretical reference for improving the buckwheat yield and quality. In this study, perennial tartary buckwheat DK19 was used as the experimental material, its key metabolic pathways in the response to Cd stress were identified and verified through transcriptomic and metabolomic data analysis. In this investigation, 1798 metabolites were identified through non-targeted metabolomic analysis containing 1091 up-regulated and 984down-regulated metabolites after treatment. Kyoto Encyclopedia of Genes and Genomes (KEGG) analysis of differential metabolites was significantly enriched in galactose metabolism, glycerol metabolism, phenylpropane biosynthesis, glutathione metabolism, starch and sucrose metabolism. Linkage analysis detected 11 differentially expressed genes (DEGs) in the galactose metabolism pathway, 8 candidate DEGs in the lipid metabolism pathway, and 20 candidate DEGs in the glutathione metabolism pathway. The results of our study provided useful clues for genetically improving the resistance to cadmium by analyzing the molecular mechanism of cadmium tolerance in buckwheat.

## 1. Introduction

Heavy metals affect the physiological metabolism of plants growing in polluted soil, and their harm is becoming increasingly prominent with the development of the economy. The accumulation of heavy metals in plants can inhibit growth and development, leading to yellowing, aging, curling, withering, decay, and even death of plant leaves [1]. Cadmium (Cd) is a highly toxic heavy metal that widely exists in both natural and artificial environments, it has toxic effects on plants growing. The heavy metal Cd present in the environment can pose a serious threat to human health through the food chain [2,3]. After plants absorb cadmium, a portion of it remains in the roots and cannot be transported to the aboveground. Cadmium absorbed by the root will combine with organic acids and amino acids in the xylem duct and be transported to the upper ground along the xylem. Stress response is generated by regulating multiple biological processes, such as cadmium fixation of cell wall, chelation of plant chelating peptide, compartmentalization of vacuoles, combination of extracellular secretions with Cd^2+^, Anabolism of sulfur and activation of antioxidant mechanism, which affect normal growth and development of plants [4,5].

To obtain high-resistance crop varieties with Cd resistance, we must first identify the genes or proteins that respond to Cd stress, and then determine the Cd resistance and detoxification mechanisms of these targeted genes or proteins [6,7]. Transcriptome analysis was used to analyze the response of plants to cadmium stress, and identification of potential differentially expressed genes was gradually carried out [8,9]. Candidate genes were involved in cell wall biosynthesis, glutathione (GSH) metabolism [10], detoxification [11], vacuole isolation [12], the antioxidant system [13], tricarboxylic acid (TCA) cycle, and these processes may play pivotal roles in cell wall binding [14]. With the development of molecular biology, research on the molecular mechanisms of plant response to cadmium stress has been found that the cadmium uptake process in plants is regulated by genes related to cadmium transport and accumulation [15]. The enrichment characteristics of heavy metal cadmium in plants are related to various traits, including root absorption ability, root to stem migration ability, and aboveground accumulation ability [16]. The key genes of the cadmium stress responsive transport system in plants include the natural resistance associated macrophage protein (NRAMP) family related to natural resistance [17], the heavy metal ATPase (HMA, heavy metal ATPase) family [18], as well as the zinc regulated transport protein and iron regulated transport protein (ZIP, Zrt-/Irt like protein) family [19] and some plant specific antioxidant enzyme genes [20].

Metabolite analysis has been used to study the response of plants to diverse stresses, including nutrient deficiency [21], mineral toxicity [22], temperature [3], oxidative stress [23], and osmotic stress [24]. The LC-MS metabolomics analysis of *Brassica napus* demonstrates that the production of reducing agents plays an important role in promoting detoxification mechanisms and stabilizing membranes [25]. After cadmium stress treatment, 12 metabolites related to amino acid synthesis (valine, leucine and isoleucine) were identified in rice through metabonomic analysis [26]. The metabolomics analysis of rice grains under cadmium stress also indicates that the metabolism of carbohydrates, organic acids, and amino acids is significantly affected [27]. Transcriptomic studies of gene expression, regulatory systems, and regulatory network changes at the mRNA level can elucidate the overall changes in endogenous metabolic substances in plant organisms, help identify the deep relationship between metabolites and corresponding changes in the physiology and pathology in the body.

*Fagopylum tatarium* (L.) Gaertn also known as bitter buckwheat or kuqiao, is an important food and medicinal crop with high nutritional value. In recent years, buckwheat has garnered global attention because of its rich nutritional and medicinal value, adaptability to poor soil conditions, and high production value and potential [28]. Since the first published reference genome, the related characteristics of Tartary buckwheat, family analysis, and omics analysis have been gradually developed. Zhang et al. predicted several new genes involved in rutin biosynthesis and regulation, aluminum stress resistance [29,30], salt stress resistance [31], drought stress resistance [32] and cold stress responses [33]. The mapping of increasingly rich expression profiles based on transcriptome data from diverse tissue stages and Tartary buckwheat treatments has greatly enhanced our understanding of life mechanisms and specific biological traits.

Buckwheat is mainly cultivated in hilly and mountainous areas, with significant overlap with metal contaminated areas. Perennial roots also exacerbate its enrichment of cadmium and other metal ions. Analyzing the cadmium response mechanism of buckwheat and cultivating resistant materials have important theoretical and practical potential. Currently, except for a few studies on buckwheat responses to cadmium stress using transcriptome analysis, studies on buckwheat under cadmium stress mainly focus on physiological characteristics [34]. The accumulation of metabolites is the final manifestation of biological response, and the analysis of metabolome is the in-depth study of buckwheat cadmium resistance. This study deeply analyzes the cadmium stress response of buckwheat through the conjoint analysis of Transcriptome and metabolome, providing a potential genetic basis for further resistance breeding.

## 2. Materials and Methods

### 2.1. Plant Material and Cadmium Treatment

The samples used in this study were obtained from the College of Taiyuan Normal University (Taiyuan, China). The experiment was conducted in greenhouse at Wuhan Polytechnic University (Wuhan, China) using the planting method.

Seeds of the duoku19 (DK19) with similar size were germinated on wet filter paper in a germination box in greenhouse cultivation. The seedlings were transplanted into Hoagland nutrient solution at the greenhouse cultivation conditions as: 16 h of light at 25–30 °C and 8 h of darkness at 15–18 °C, and 400 μmolm^−2^ s^−1^ intense luminosity. The buckwheat seedlings with 7–8 leaves at the consistent growth were treated with Cd. Buckwheat planted in the Cd solution (10 μ Mol/L, cadmium source CdCl_2_·2.5H_2_O). After 6 h of treatment, the material taken out as Cd—6h, and after 24 h of treatment, the material taken out as Cd—24h, the material without cadmium treatment was CK. Three treatments were set up, exchanged and absorb cadmium with 20 mmol/L Na_2_-EDTA from the root surface, and washeed with deionized water [35].

### 2.2. Determination of Oxidative Biomarker and Antioxidant Enzyme Activity

The oxidative biomarker and antioxidant enzyme activity indices of each treatment were measured three times. The malondialdehyde (MDA) content was determined using thiobarbituric acid TBA [36]. Fresh leaves (0.5 g, three replicates) were taken in liquid nitrogen precooled mortar and ground them to powder. The powder was placed in to 8 mL precooled 0.05 mol L^−1^ sodium phosphate buffer (pH 7.8). Centrifuged at 10,000 rpm for 20 min and extracted the supernatant for enzyme activity measurement at 4 °C for enzyme activity determination. Peroxidase (POD) activity was determined using the guaiacol method [37]. Sample preparation, extraction, and storage were similar to MDA, except for different detection methods. The activity of superoxide dismutase (SOD) was determined using NBT photoreduction method [38]. 0.5 g fresh leaves were taken in liquid nitrogen precooled mortar and ground them to powder. The powder was placed in to 5 mL precooled sodium phosphate buffer (pH 7.8). Centrifuged at 10,000 rpm for 20 min and extracted the supernatant for enzyme activity measurement at 4 °C to measure absorbance. The activity of catalase (CAT) was determined using the TBA-TCA method [35]. Fresh leaves (0.5 g, three replicates) were taken in liquid nitrogen precooled mortar and ground them to powder. The powder was placed in to 8 mL precooledsodium phosphate buffer. Centrifuged at 10,000 rpm for 20 min and extracted the supernatant for enzyme activity measurement at 4 °C for enzyme activity determination.

### 2.3. Untargeted Metabolomics of Buckwheat Leaf Tissues

Three experimental replicates performed at each treatment stage, and 10 samples with consistent growth were fully crushed in liquid nitrogen ground to powder in a ball mill (MM400, Retsch, Germany) at 30 Hz for 45 s. 0.05–0.1 g powdered sample was extracted and performed using a Perkin Elmer 680 GC (Perkin Elmer Inc., Akron, OH, USA) and Q Exactive Focus Orbitrap LC-MS/MS (Thermo Scientific, Waltham, MA, USA) [39].

The recorded data were processed with compound discoverer (CD) 3.1 software to obtain the mass-to-charge ratio, retention time, and MS/MS2 information of all detected substances. The accumulated content of metabolites is calculated through fluid coverage for later analysis. The correlation coefficient between materials was calculated using R software package ropls and detected through Principal component analysis (PCA). Through metabolite screening, differentially accumulated metabolites (DAMs) with fold change (FC) criteria > 1.50, *p*-value < 0.05 were considered to determine significant differences between samples. KEGG pathway enrichment analysis was performed using R using data from http://www.genome.jp/kegg/, accessed on 21 May 2023, respectively.

### 2.4. RNA Extraction and High Throughput Transcriptome Analysis

Total RNA was extracted and the libraries were constructed using TruSeq Stranded mRNA LTSample Prep Kit (Illumina, San Diego, CA, USA) according to the manufacturer’s instructions and sequenced using Illumina Hi-Seq platform [40]. Transcripts Per Million mapped reads (TPM) value of each gene was obtained by htseq-count. DEGs were identified using the DESeq package function sestimate Size Factors and nbinomTest. *p* value < 0.05 and fold Change > 2 or fold Change < 0.5 was set as the threshold for significantly differential expression. Gene Ontology (GO) enrichment and KEGG pathway enrichment analysis of DEGs were respectively performed using R [41].

## 3. Results

### 3.1. Determination of Oxidative Biomarker and Antioxidant Enzyme Activity

**Figure 1:**
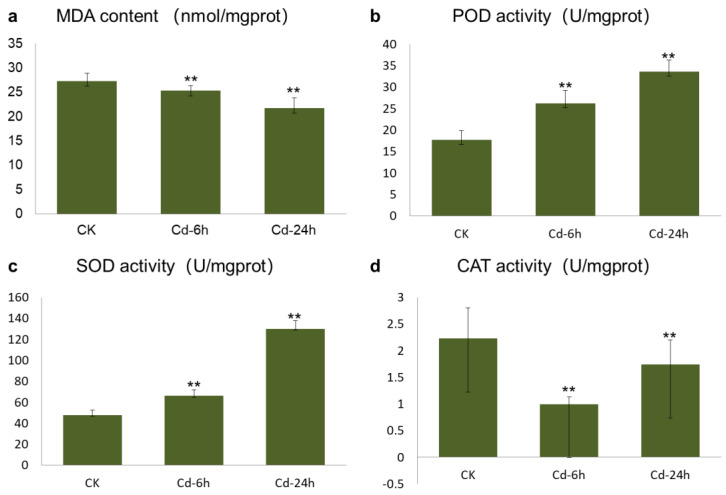
Determination of oxidative biomarker and antioxidant enzyme activity of buckwheat leaves under Cd stress. (**a**) MDA content. (**b**) POD content. (**c**) SOD content. (**d**) CAT content. The *x*-axis in the column chart represented different treatment stages, with CK representing untreated tissue, Cd—6h representing tissue treated for 6 h, and Cd—24h representing tissue treated for 24 h. The y-axis represented the content of different contents Significance levels: ns, not significant (*p* > 0.05), ** *p* ≤ 0.01.

Malondialdehyde (MDA) is the final product of membrane lipid peroxidation, and its content can directly reflect the toxicity of Cd to plants. The increase in MDA content reflects an increase in the concentrations of -OH, O_2_^−^, and other free radicals in the body to a certain extent. Figure 1a showed that the MDA content in buckwheat leaves decreased with increasing Cd treatment times, and the difference between the control versus Cd—6h treatment and the control versus Cd—24h treatment was very significant (*p* < 0.01). The promotion rates after 6 and 24 h of treatment were 25.253 and 21.724 m mol/g, respectively. The decrease in MDA content was caused by the increase in antioxidant enzyme activity in plants induced by Cd and the timely removal of excess -OH, O_2_^−^, and other free radicals.

Figure 1b showed that the peroxidase (POD) activity of buckwheat DK19 significantly increased after 6 h of treatment (*p* < 0.05), when the Cd stress time was extended to 24 h, the POD activity was significantly lower than that in the untreated or 6-h Cd treatment group (*p* < 0.05). The activity of superoxide dismutase (SOD) in DK19 leaves increased with prolonged treatment under external Cd stress (Figure 1c). The difference in SOD activity between the two treatments was statistically significant (*p* < 0.05). After 6 h of treatment, the catalase (CAT) activity in buckwheat leaves decreased compared to that in the treatment without Cd, and significantly decreased between treatments (*p* < 0.05) (Figure 1d). This trend was maintained throughout 24 h of treatment.

### 3.2. Statistical Description of Metabolic Analysis of Untargeted Metabolomics Sequencing

**Figure 2:**
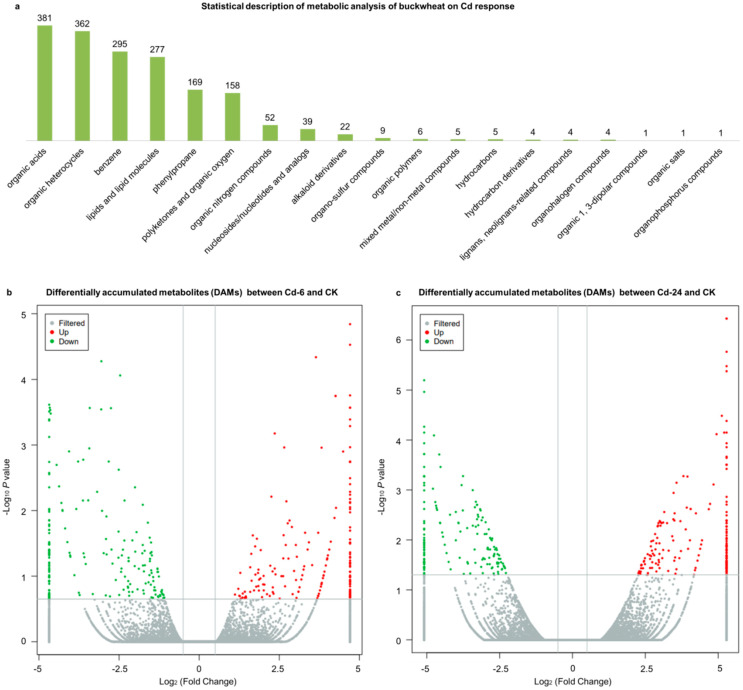
Statistical description of untargeted metabolomics sequencing. (**a**) The ratio of detected metabolites to total metabolites in the column chart. The abscissa and ordinate represent the grouping and type of metabolites, respectively. (**b**) The volcano plot showed the DAMs between the 6-h treatment (Cd—6) and control (CK) groups. (**c**) The volcano plot showed the DAMs between the 24-h treatment (Cd—24) and CK groups. The horizontal and vertical axes represent the Log2 (fold change) and—Log10p values; the red and green dots represent up-regulated and down-regulated metabolites.

Through metabolite screening, a total of 1798 metabolites were identified. All the metabolites were divided into 19 categories (Figure 2a), wherein 68 classes were identified. Differentially accumulated metabolites (DAMs) were considered to determine significant differences between samples. A total of 479 up-regulated and 406 down-regulated metabolites were observed between the 6-h treatment (Cd—6) and control (CK) groups (Figure 2b). In the second treatment period (24 h), 612 up-regulated metabolites and 578 down-regulated metabolites were observed, respectively (Figure 2c). Corresponding to the observed phenotypic variation, the metabolism in buckwheat cells changed dramatically under Cd stress.

**Figure 3:**
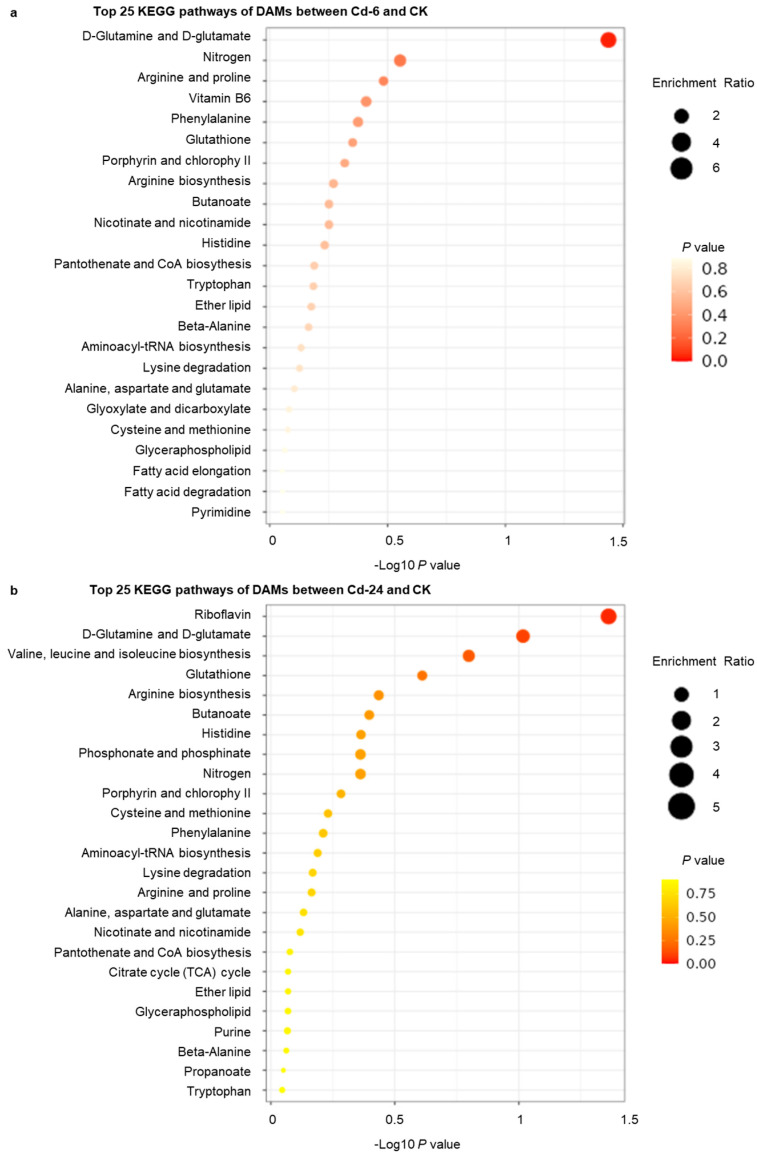
The top KEGG pathways of all DAMs between different treatment stages. (**a**) The top 25 KEGG pathways of DAMs between Cd—6 and CK. (**b**) The top 25 KEGG pathways of DAMs between Cd—24 and CK. The darker the color, the lower the *p* value. The larger the diameter of the dot, the more number of genes.

KEGG enrichment analysis of DAMs was conducted, and the 25 top pathways were shown in Figure 3. In the first stage of treatment, the pathways that were enriched the most were phenylalanine, riboflavin metabolism, tyrosine, vitamin B6 metabolism, and nicotinate and nicotinamide metabolism pathways (Figure 3a). Figure 3b was shown the top 25 KEGG pathways in the second treatment period. The top five enriched KEGG pathways were phenylalanine metabolism, purine metabolism, tyrosine and tryptophan metabolism, riboflavin metabolism, and glutathione metabolism, considering the materials before treatment as the control.

### 3.3. Statistical Description of Transcriptome Analysis

**Figure 4:**
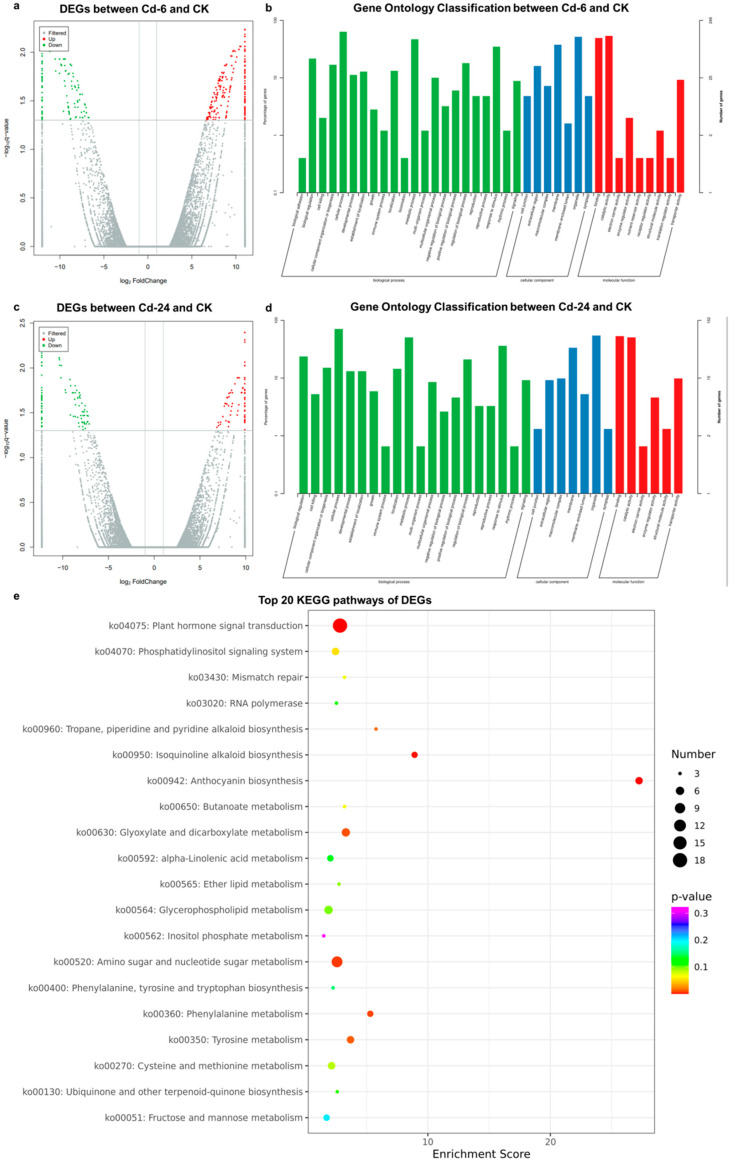
Statistical description of DEGs of high-throughput transcriptome analysis. (**a**) The volcano plot presents the DEGs between the Cd—6 and CK groups. (**b**) Top 25 gene ontology classification between the Cd—6 and CK groups (**c**) The volcano plot shows the DEGs between the Cd—24 and CK groups. (**d**) Top 25 gene ontology classification between the Cd—24 and CK groups. (**e**) Top 20 KEGG pathways of DEGs. The darker the color, the higher is the *p* value. The larger the diameter of the dot, the more abundant the number of genes. In the volcanic map, the horizontal and vertical axes represented the Log2 (fold change) and—Log10p values, respectively; the red and green dots represent the up-regulated and down-regulated metabolites, respectively. In the gene ontology (GO) classification analysis, The abscissa and ordinate represent the name of the GO unit and the number of genes contained, respectively. The green column belongs to the biological process, the blue column belongs to the cellular component, and the red column belongs to the molecular function.

High-throughput transcriptome analysis was conducted using the same materials as the metabolic analysis, and a total of 73.37 GB of clean data was obtained. We observed 286 and 171 up-regulated and down-regulated genes after 6 h of treatment (Figure 4a), and 221 up-regulated and 190 down-regulated genes after 24 h of treatment (Figure 4c), respectively. All unique DEGs were divided into biological process (Figure 4b,d green), cellular component (Figure 4b, 4 blue), and molecular function (Figure 4b, 4 red) categories in gene ontology (GO) classification analysis. The top GOs after 6 and 24 h of treatment are shown in Figure 4b,d, respectively. At 6 h, five GO units, namely, cellular processes (containing 158 genes), metabolic processes (containing 117 genes), responses to stimuli (containing 87 genes), and biological regulation (containing 54 genes), belonged to biological processes, with the most enriched genes. Five GO units, namely, organelle (containing 129 genes), membrane (containing 94 genes), extracellular region (containing 40 genes), macromolecular complex (containing 18 genes), and cell junction (containing 12 genes), belonged to the cellular component, with the most enriched genes. Catalytic activity (containing 134 genes) and binding (containing 123 genes) were the two GO units with many genes enriched in molecular functions. Between the 24-h treatment (Cd—24) and CK groups, the secondary GO units with the most significant enrichment were cellular process, organelle, binding, metabolic process, catalytic activity, response to stimulus, membrane, biological regulation, regulation of biological processes, and cellular component organization or biogenesis. According to the KEGG enrichment analysis (Figure 4e), 523 DEGs were mainly enriched in 90 metabolic pathways, wherein pathways with significant differences in gene enrichment were biosynthesis of secondary metabolites (accounting for 21.02% of all DEGs) and metabolic pathways (accounting for 23.22% of all DEGs), followed by cellular processes and environmental information processing. The significantly enriched pathways included amino acid metabolism, lipid metabolism, energy metabolism, carbohydrate metabolism, transport and catabolism, metabolism of other amino acids, and signal transduction.

### 3.4. Analysis of the Key Metabolic Pathways Based on the Integration of Transcriptome and Metabolome

According to previous studies, the obtained differential genes were uploaded to the GO and KEGG databases, and the differences were annotated and enriched. KEGG metabolic network analysis was conducted on the above results and metabolic pathways that respond to gene expression and metabolites after Cd stress included eight pathways: galactose metabolism, α-linolenic acid metabolism, ABC transporter, glyceride metabolism, phenylpropane biosynthesis, glutathione metabolism, starch and sucrose metabolism, and cysteine and methionine metabolism.

**Figure 5:**
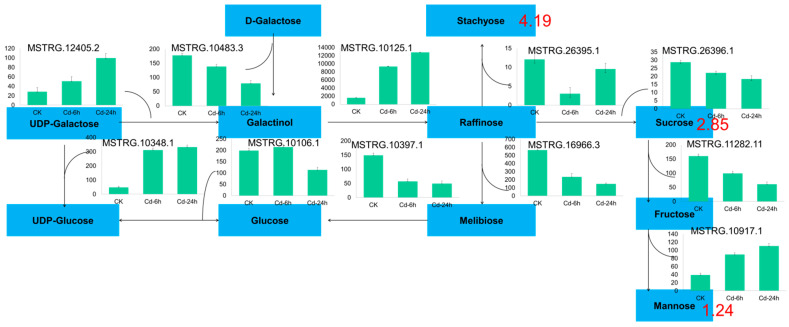
Galactosse metabolism pathway in buckwheat under Cd stress. The blue plate represented the metabolites; the green column chart represented the gene ID and expression, and the number represented the multiple of metabolites.

All carbohydrate differential metabolites showed an upward trend, including stachyose (4.19-fold), sucrose (2.85-fold) and mannose (1.24-fold). A total of 20 DEGs were enriched in galactose metabolism, 8 DEGs were enriched in fructose and mannose metabolism, and 30 DEGs were enriched in starch and sucrose metabolism. In this study, 11 selected candidate genes were tested to verify the galactose metabolic pathway, and the results are shown in Figure 5.

**Figure 6:**
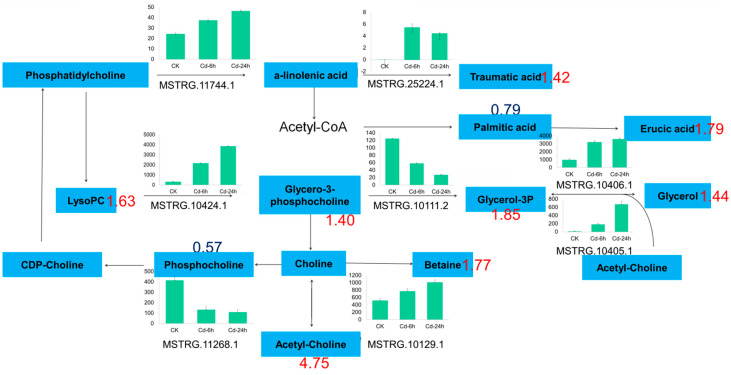
Lipid metabolism pathway in buckwheat under Cd stress. Numbers represented the fold change of metabolites. The blue plate represented metabolites; the green column chart represented the gene ID and expression; and the number represented the multiple of metabolites.

In this study, two glyceride metabolism DAMs, three glyceride metabolism DAMs, and one glyceride and α-linolenic acid metabolism DAMs were enriched in lipid metabolism pathway, Including glycerol triphosphate (1.85-fold), lecithin (1.63-fold), palmitic acid (0.79-fold), betaine (1.77-fold), etc. (Figure 6). The transcriptional showed that DEGs were 12, 12 and 14 enriched in these metabolisms. A total of nine differential genes were enriched in glyceride metabolism, 13 were enriched in glyceride metabolism, and 20 were enriched in glyceride and α-linolenic acid metabolism after 24 h of Cd stress.

**Figure 7:**
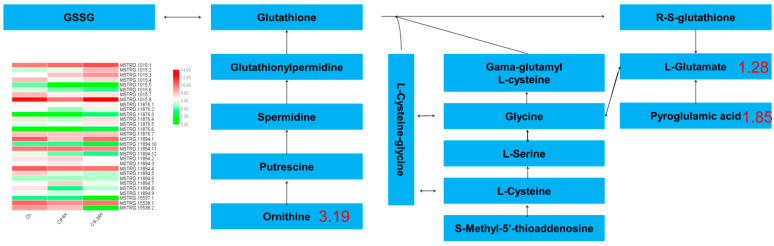
Glutathione metabolism pathway in buckwheat under Cd stress. The blue plate represented metabolites; the green column chart represented the gene ID and expression; and the number represented the multiple of metabolites.

A total of 4 DAMs were enriched in glutathione metabolism, in which 77 DEGs and 81 DEGs were enriched after 6 h and 24 h of Cd stress, respectively. The metabolites involved in glutathione metabolism included ornithine (3.19-fold), glutamic acid (1.28-fold), and pyroglutamic acid (1.85-fold), etc. A heat map of the expression ko00480 genes is shown in Figure 7, showed that 8 genes showed a continuous upward trend. With the extension of treatment time, the expression of 7 genes decreased with the treatment. 9 genes significantly decreased their expression in the first stage of treatment, and increased their expression in the second stage of treatment. Two genes first increased their expression and then decreased their expression. The expression of 4 genes did not change significantly. Changes in the expression of these genes are the main reason for the increase in ornithine and pyroglutamic acid contents and the decrease in the glutamic acid content.

## 4. Discussion

### 4.1. Effects of Cd Stress on Buckwheat

Cd pollution in farmland soils seriously threatens agricultural productivity and human health [42,43]. Cadmium pollution in plants destroys the plasma membrane composition and integrity, blocks the water metabolism, inhibits photosynthesis, reduces respiration, disrupts carbohydrate and nitrogen metabolism, and inhibits plant hormone secretion, leading to an imbalance of elements in plants and the inhibition of seed germination, nutrient growth, and plant development [44,45]. In this study, we observed that buckwheat growth was significantly inhibited after Cd treatment and it demonstrated extremely significant weakening or even death after long-term treatment. Significant changes in the MDA, POD, SOD, and CAT levels were detected in plants. The phenotypic changes and physiological reactions of buckwheat detected in this study were consistent with those in other species after Cd treatment, indicating the accuracy of this study and the feasibility of detecting changes in gene expression [46,47].

In this study, we evaluated the galactose, lipid, and glutathione metabolism, which were significantly enriched at the gene and metabolic levels, combined with transcriptome and metabolome data. These metabolic pathways have been reported in studies on indica rice, wheat, corn, and other crops. Metabolomic analysis of *Brassica napus* by LC-MS showed that reductant production plays an important role in promoting detoxification mechanisms and stabilizing membranes [25]. The response of proline and free amino acid content to the metabolic analysis of alfalfa under Cd stress is the vital in improving the resistance of alfalfa to Cd toxicity [48,49].

Transcriptomics analysis to study Cd-responsive genes have identified several DEGs, these genes are involved in several processes, including cell wall biosynthesis [50,51], the antioxidant system [52,53], TCA cycle [54]. Metabolomics can reveal the overall changes in endogenous metabolites in plant organisms in the internal and external environments, and help identify the deep-seated connection between metabolites and the corresponding changes in physiology and pathology in the body [55]. Xie et al. identified 12 metabolites significantly changed in amaranth after Cd stress treatment [56]. Sarry et al. used *Arabidopsis* as a research object and detected approximately 2000 metabolites by metabolomic analysis. They detected the regulatory genes of *Arabidopsis* related to glucose metabolism [57]. Comprehensive analysis of transcription and metabolomics of two different barley genotypes demonstrated that the β-dextran content and receptor-like protein kinase-leucine-rich repeat (RLK-LRR), heat shock protein (HSP), and other key genes regulate the malt quality traits and drought tolerance mechanisms [58,59].

### 4.2. Effects of Cd Stress on Plant Antioxidant System

When plants are stressed, excessive ROS are produced and accumulate in cells. When the ROS content exceeds a certain threshold, the balance of plant cells is disturbed, the cell membrane is damaged, and lipid peroxidation occurs. Damage to membrane integrity damages the membrane system and cell oxidation. The resistance of plants to Cd stress depends on their ROS-scavenging capacity, and plants maintain ROS balance through an effective antioxidant system [60,61,62]. Results have demonstrated that with an increase in Cd stress concentration and time, the maize leaf antioxidant system was seriously damaged, and the active oxygen metabolism in the leaves was disordered. High concentrations of Cd severely damaged the maize leaf membrane system [63]. Therefore, in addition to the higher lipid peroxidation intensity, the level of antioxidant enzymes in plants severely stressed by Cd also decreases, and the root, stem, and leaf tissues of plants are damaged, resulting in the death of affected plants.

GSH is a non-protein mercaptan that has been proved to be an important antioxidant and is critical for oxidation-reduction homeostasis. Several studies have demonstrated that plants respond to oxidative stress by producing excessive amounts of antioxidants (including GSH, ascorbic acid, and polyphenols). GSH predominantly removes heavy metals from plants in two ways [64,65]. First, GSH can directly capture and combine heavy metal ions on the sulfur base of the enzyme protein to achieve detoxification. GSH is a precursor for plant chelatin synthesis. An increase in its content accelerates plant chelatin synthesis, facilitating its chelation with heavy metals [66]. Under the action of the corresponding transport proteins, they are transported to vacuoles or excluded from the cell. Researches have shown that when plants respond to Cd stress, they increase the activity of antioxidant enzymes, activate the up-regulated genes involved in the ascorbic-glutathione cycle, and increase glutathione, ascorbic acid, and plant chelating protein content to alleviate oxidative stress and Cd-induced growth inhibition and reduce Cd toxicity in plants [67,68].

Glutamic acid, ornithine, and proline metabolism are the major nitrogen metabolism pathways in plants [69,70,71]. In this study, the ornithine and pyroglutamic acid contents increased, respectively, and the glutamic acid content decreased. According to the transcriptome data, they also encode glutathione hydrolase Gamma—. The Gamma —glutamyltransferase (ggt) gene of glutamyl transpeptidase was downregulated by 1.57-fold. Gamma—Glutamate, the metabolite regulated by glutamyltranspeptidase, demonstrated a downward trend; however, L-cysteine-glycine regulated by glutathione hydrolase did not change significantly, possibly because glutathione metabolism was only a small part of glutathione through ggt gene expression. Moreover, under Cd stress, the OXP1 gene encoding hydroxyprolinase in buckwheat showed a downward trend, which may explain the decrease in glutamate content.

### 4.3. Response of Galactose Metabolism to Cd Stress

When plants are under abiotic stress, they induce gene expression to synthesize small molecular compounds with osmotic regulation, thus maintaining normal metabolism and plant growth [72]. Previous studies have shown that carbohydrate accumulation in plant tissues is to regulate cellular osmotic pressure and maintain osmotic balance to adapt to environmental changes under abiotic stress [73]. Under high heavy metal concentrations, plants also demonstrated a similar accumulation of carbohydrates in their roots, which can be speculated to have certain similarities with other stress response mechanisms in plants under Cd stress; namely, stachyose, sucrose, and mannose content increased in buckwheat [74]. Sugar content of rice seeds under Cd stress demonstrated the same trend [75]. Two genes encoding galactosyl synthetase 2 play a key role in galactosyl synthesis under Cd stress, and their activities determine the raffinose oligosaccharide content, which plays an important role in the response of plants to abiotic stress. In conclusion, buckwheat may improve its adaptability to Cd stress through the accumulation of carbohydrates, including sucrose and galactose metabolism. The major reason for carbohydrate accumulation is the significant upregulation of galactosyltransferase gene expression. Galactol synthetase (Gols), a key enzyme in raffinose oligosaccharide synthesis, plays an osmoregulatory role in plant cells [76,77].

### 4.4. Response of Lipid Metabolism to Cd Stress

The stress response under abiotic stress increased the glycitol content, including the alanine and glycerol contents, and maintained the unsaturated fatty acid content in chloroplasts, which aided in improving the resistance of plants to abiotic stress [78]. In this study, Cd stress increased the levels of metabolites, including lecithin, callus acid, glycerol, and palmitic acid. These fatty acid derivatives contain α- and β-unsaturated carbonyls, which are reactive electrophilic species that alleviate heavy metal stress by activating the detoxification mechanism and stress response, including HSP and ATP-binding cassette (ABC) transporters. According to the transcriptome data, the open reading frame (OPR) of the gene encoding 12-oxo-phytodienoic acid (PDA) reductase in buckwheat increased by 4.89-fold under Cd stress, which may be related to membrane lipid peroxidation mediated by oxidative stress under Cd stress. According to the results of transcriptomic and quantitative analyses, the expression of phospholipase (phospholipases A and A2) and lysophospholipase I (LYPLA) genes increased under Cd stress, which may explain the increase in the lecithin and glycerol-3-phosphate choline contents. The increase in the content of oxygen-containing fatty acids indicates that buckwheat may use its lipid signal to regulate protective or defense mechanisms. Therefore, the above results indicated that the lipid metabolism-related pathway may participate in Cd stress and tolerance mechanisms.

## 5. Conclusions

In this study, the perennial tartary buckwheat variety DK19 was utilized as the test material, and combined with transcriptome sequencing and non-targeted metabonomics technology, we explored the physiological and response mechanisms under Cd stress. In this study, we integrated and evaluated transcriptome and metabolomic data, and identified multiple enrichment pathways, including galactose, amino acid, lipid, glutathione, phenylpropane, cysteine, and methionine metabolism. We focused on galactose, glutathione, and lipid metabolism and identified some upregulated or downregulated genes. These results demonstrated that mechanism of buckwheat under Cd stress was governed by a complex regulatory and signaling mechanism. Through the analysis of existing data, we have deepened our understanding of buckwheat’s response to cadmium stress and predicted some potential genes, providing a basis for further analysis of cadmium stress resistance.

## Data Availability

The dataset and materials presented in the investigation is not publicly available due to privacy concern but will be available from the corresponding author.
